# Comparative evaluation of two dural closure techniques for U-shaped incisions: sealing efficacy vs. site-specific infection risk

**DOI:** 10.3389/fsurg.2025.1728951

**Published:** 2026-01-12

**Authors:** Yu Wang, Chang-Zhi Zhao, Kai-Long Zhang, Yi-Xiao Chen, Ao Li, Jie Yin, Fei Long, Qi-Hong Wang

**Affiliations:** 1 Xuzhou Clinical School of Xuzhou Medical University, Xuzhou, China; 2 School of Health Science and Engineering, University of Shanghai for Science and Technology, Shanghai, China; 3 Department of Neurosurgery, Xuzhou Central Hospital, Southeast University, Xuzhou, Jiangsu, China; 4 The 904th Hospital of PLA, Medical School of Anhui Medical University, Wuxi, China

**Keywords:** autologous fascia, cerebrospinal fluid leak, dural repair, neurosurgical procedures, surgical site infection

## Abstract

**Objective:**

This study aimed to compare the outcomes of direct suturing (Method 1) and pericranium-assisted suturing (Method 2) for U-shaped dural incisions, with a specific focus on site-specific differences in cerebrospinal fluid (CSF) leak and postoperative infection.

**Methods:**

In this retrospective cohort, 172 patients undergoing repair of U-shaped dural incisions were analyzed. Based on intraoperative feasibility, patients underwent either Method 1 (*n* = 94) or Method 2 (*n* = 78). Primary and secondary outcomes were CSF leak and postoperative infection rates, respectively. Subgroup analyses were stratified by surgical site (supratentorial vs. infratentorial).

**Results:**

The incidence of CSF leak was low and comparable between the two methods, regardless of surgical site (Method 1: 7.14% supratentorial vs. 7.69% infratentorial, *P* = 1.00; Method 2: 4.17% vs. 3.33%, *P* = 1.00). Re-repair rates were similarly low across all groups. However, Method 2 was associated with a significantly higher overall infection rate in the infratentorial compartment compared to supratentorial surgeries (23.33% vs. 6.25%, *P* = 0.039). Sub-analysis revealed this was primarily driven by a higher incidence of incision infection/delayed healing in the infratentorial group (16.67% vs. 2.08%, *P* = 0.028), whereas meningitis rates were comparable. Multivariable analysis confirmed the surgical site itself was not an independent risk factor for infection.

**Conclusion:**

Both direct and pericranium-assisted suturing are effective in preventing CSF leak for U-shaped dural incisions. However, the pericranium-assisted technique carries a significantly increased risk of incision-related infections in the infratentorial region. Clinical decision-making must therefore balance the reliable sealing capability of pericranium-assisted repair against its site-specific infection profile, particularly in complex posterior fossa surgeries.

## 1 Introduction

Dural closure is a critical step in most cranial surgeries. However, no unified consensus currently exists regarding the standardization of dural repair procedures. Previous studies indicate that clinicians often prefer to reinforce primary suturing with sealants or dural substitutes to achieve watertight closure, with satisfactory clinical outcomes reported (1–5). The shape of dural incisions varies, commonly including “U-shaped,” “Y-shaped,” and “L-shaped,” influenced by factors such as surgery type and location. The choice of incision type and repair method often depends on the surgeon's experience and preference, compounded by the diversity of repair materials (e.g., sutures, sealants, dural substitutes) (6, 7), which poses challenges to establishing consistent clinical practices. Currently, the comparative clinical benefits of different systematic repair approaches remain inconclusive, and robust research in this area is still limited (8). Achieving watertight dural closure is paramount, and suturing technique is crucial (9). However, intraoperative factors like dehydration, light exposure, and electrocautery can cause dural shrinkage, significantly increasing the difficulty of direct suturing and elevating the risk of CSF leak and other complications (10). This study therefore introduces two distinct repair protocols for the common U-shaped dural incision—direct suturing and fascia-assisted suturing—the choice of which is determined by the intraoperative feasibility of direct dural closure. The aim is to analyze the clinical efficacy of these two approaches in supratentorial and infratentorial surgeries, compare their applicability, provide references for clinical practice, and systematically evaluate their outcomes in different surgical contexts.

Autologous fascia, as a traditional autograft, is widely used due to its advantages of being non-toxic, non-immunogenic, and capable of vascularization (7, 11). Its unique biomechanical properties are also reasons for its popularity (12, 13). Pericranium, a type of autologous fascia, compared to fascia lata, does not require an additional surgical incision, making it an ideal repair material. Current reports on pericranium application predominantly focus on infratentorial and posterior fossa surgeries (5, 14, 15), with relatively fewer reports for other sites. One of the repair methods evaluated in this study utilizes pericranium as an auxiliary material, aiming to compare whether the repair technique incorporating pericranium yields satisfactory results for the same type of U-shaped incision in both supratentorial and infratentorial locations**.** The second part of this study involves a systematic review of recent literature on the application of autologous fascia in duraplasty, aiming to corroborate our findings and provide an in-depth discussion on the clinical outcomes of pericranium as a traditional dural substitute at different surgical sites.

## 2 Methods and materials

### 2.1 Study design and patient cohort

This single-center retrospective cohort study received approval from the institutional ethics committee (Approval No.: XZXY-LK-20250508-0050). Informed consent was waived due to the use of retrospective anonymized data. We systematically screened patients who underwent cranial surgery requiring dural repair at our center between January 2020 and January 2025. Inclusion criteria were: 1) First-time dural repair surgery; 2) Surgical records clearly describing the dural incision as “U-shaped”. Exclusion criteria were: 1) Spinal dural repair surgery; 2) Non-primary repair surgery; 3) Dural repair not performed due to procedures like decompressive craniectomy; 4) Incomplete medical records; 5) Poor postoperative compliance, unable to complete follow-up; 6) Non-U-shaped dural incision (e.g., “L-shaped,” “Y-shaped,” etc.); 7) Age <18 years. Ultimately, 172 patients were included in the analysis. Baseline data, surgical records (including dural incision characteristics, dural repair time, method), and postoperative complications were collected via the hospital medical record system. All patients received strict perioperative management, including preoperative comprehensive examinations, postoperative intensive monitoring (low intracranial pressure positioning, routine intracranial pressure reduction, nutritional support, prophylactic antibiotics, wound care). All patients underwent at least one imaging examination before discharge and were instructed to return for follow-up at 1 month, 3 months, and 6 months postoperatively. All patients completed at least 6 months of follow-up.

Outcome measures: Cerebrospinal fluid leakage, defined as any of the following conditions: 1) cerebrospinal fluid leakage or epidural effusion detected by imaging during hospitalization or follow-up; 2) postoperative persistent orthostatic headache; 3) Patients were readmitted due to cerebrospinal fluid leakage and underwent surgical repair. Postoperative infection, classified as deep meningitis, was determined on the basis of the presence of a pathogen in a cerebrospinal fluid culture or direct examination and was accompanied by at least one of the following symptoms: fever, meningeal irritation, neck stiffness, or general irritability; Superficial infection; The surgical incision was suppurated and delayed healing. Pseudomeningocele: assessed by radiographic imaging or observed during subsequent surgery.

### 2.2 Incision classification and repair surgical protocol

All surgeries were performed by the same senior neurosurgical team (one chief physician and two associate chief physicians) to minimize operator variability. The dural repair protocol was discussed and established by the team. The general indications for harvesting pericranium to assist in repair included; 1. Intraoperative need for extensive dural resection; 2. Discovery of an obvious dural defect during repair; 3. Failure of Method 1 repair; however, the decision was not absolute and was based on the specific intraoperative situation. After completing the main intracranial procedure, positive end-expiratory pressure (PEEP) was increased to 15 cmH_2_O and maintained for 20 s to assess hemostasis in the surgical field and dural tension and integrity.

Applicability criteria for Method 1 (Direct Suturing): After the PEEP test, assessment confirmed no significant dural shrinkage, no severe defect, the dural edges could be approximated without tension (specifically, the gap resulting from dural retraction was ≤3 mm and could be closed without undue traction). Subsequently, under microscope, the dural incision was continuously sutured with a locked stitch using 5-0 Prolene suture. After carefully coagulating bleeding points on the dural surface, biological fibrin glue (Compant®) was applied along the suture line to seal potential micro-leaks. Finally, trim the artificial dura mater to an appropriate size and completely cover the defect of the dura mater.

Applicability criteria for Method 2 (Pericranium-Assisted Suturing): After the PEEP test, the surgeon judged that the dura was significantly shrunk due to factors like dehydration or electrocautery (defined as: dural edge retraction, forming a gap width >3 mm that could not be approximated without tension). At this point, the patient's autologous pericranium was harvested and trimmed according to the shape and size of the dural defect. Similarly, under microscope, the trimmed pericranium was continuously sutured with a locked stitch to the dural edges using 5-0 Prolene suture. The subsequent steps for filling in the biological fibrin glue(Compant®) and artificial hard membrane are the same as above.

### 2.3 Literature review strategy

A systematic literature search was conducted in the PubMed database. Core search terms included (“dural repair” OR “dural reconstruction” OR “duraplasty”), with a focus on literature involving free temporal muscle fascia (FTFG), pericranium, autografts, fascia lata, etc. Literature types included case reports, clinical studies, reviews, and systematic reviews, primarily focusing on studies published in the last 5 years, followed by content organization and summary.

## 3 Statistical analysis of data

Data analysis was performed using IBM SPSS Statistics 27 software. Continuous variables are expressed as mean ± standard deviation, and intergroup comparisons were made using Student's *t*-test; categorical variables are expressed as frequency and percentage, and intergroup comparisons were made using the Chi-square test or Fisher's exact test. Additionally, to adjust for baseline differences, multivariable logistic regression models were constructed for the surgical method and primary/secondary outcomes. Statistical significance was set at *P* < 0.05.

## 4 Results

### 4.1 Patient baseline characteristics and outcome comparison

**Figure 1:**
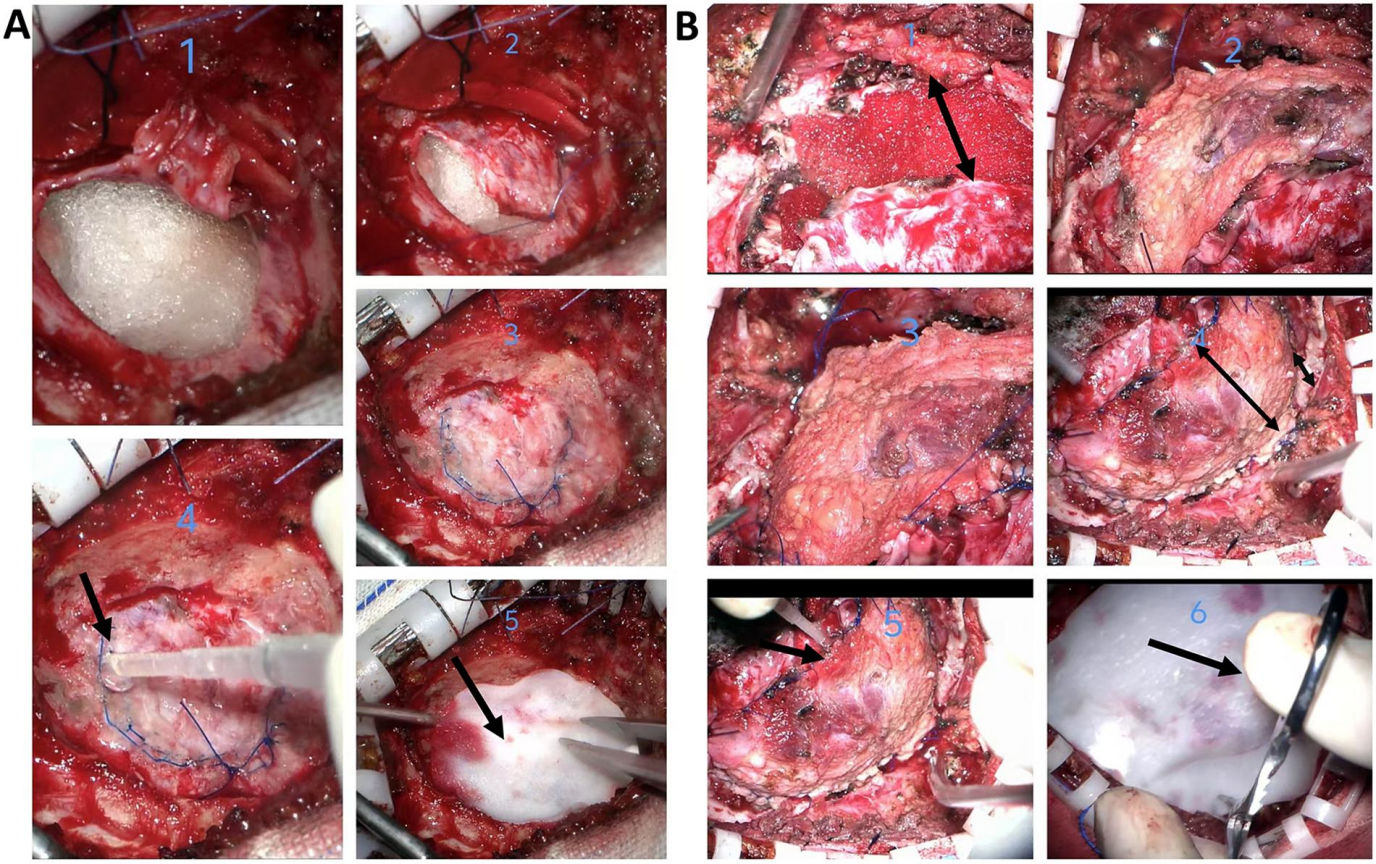
Illustrative cases of the two dural repair techniques (A: method 1, B: method 2). Arrows (→) indicate sites for biological glue application or artificial dura mater coverage. Double-headed arrows (↔) delineate the extent of the dural defect or the graft coverage area. **(A)** For Infratentorial meningioma, following tumor resection, minimal wrinkling of the dura was observed. Repair was performed using Method 1. As shown in Figure 1, the edges of the dura were approximated and continuously sutured using a locked-edge technique. Figures 2, 3, and 4 illustrate the application of biological glue (Compant®) along the dural suture line. In Figure 5, an appropriately sized artificial dural membrane was used to cover the incision site **(B)** For Infratentorial gliomas, after tumor resection, significant wrinkling of the dura was noted. Repair was carried out using Method 2. As depicted in Figure 1, The dura does not adhere closely to the edge of the incision, and the size of the gap is as shown in the annotation. The outer skull membrane was excised, trimmed to an appropriate size, and used as a patch for the incision, as demonstrated in Figures 2 and 3. Figure 4 shows the appearance after tight suture closure. The two annotations represent the range covered by the fascia for repair and the original coverage range of the dura, respectively. In Figure 5, biological glue (Compant®) was applied along the dural suture line, and Figure 6 illustrates the use of an appropriately sized artificial dural membrane for coverage.

This study included 172 patients undergoing repair of U-shaped dural incisions. Based on intraoperative dural condition (whether direct suturing was possible), they were repaired using Method 1 (Direct Suturing, *n* = 94) or Method 2 (Pericranium-Assisted Suturing, *n* = 78). The two groups were overall balanced in demographics and baseline diseases, with no significant differences in gender, age, weight, height, BMI, hypertension, diabetes, coronary artery disease, epilepsy, and cerebral infarction, or lumbar drain (LD) use (all *P* > 0.05), details in Table 1. Overall, there was no statistical difference in the incidence of the primary outcome between the two methods (Method 1: 7.45% vs. Method 2: 3.85%, *P* = 1.000). However, site-specific analysis revealed different trends. Among the 94 patients repaired with Method 1 (42 supratentorial, 52 infratentorial): The CSF leak rate was 7.14% for supratentorial surgeries and 7.69% for infratentorial surgeries, with no significant difference (*P* = 1.00). The re-repair rate was 4.76% for supratentorial and 0% for infratentorial surgeries (*P* = 0.383), the infection rate was 7.14% for supratentorial surgeries and 1.92% for infratentorial surgeries, with no significant difference (*P* = 0.464). When further categorizing infection types, the incidence of incision infection/delayed healing was 2.38% vs. 0% (*P* = 0.914), and meningitis was 4.76% vs. 1.92% (*P* = 0.851) for supratentorial and infratentorial sites, respectively. The incidence of pseudomeningocele showed no significant difference between supratentorial and infratentorial sites (2.13% vs. 0%, *P* = 0.571). Among the 78 patients receiving Method 2 (48 supratentorial, 30 infratentorial): The CSF leak rate was 4.17% for supratentorial and 3.33% for infratentorial, with no significant difference (*P* = 1.00). Re-repair was performed in 4.17% of supratentorial cases and 0% of infratentorial cases (*P* = 0.692). The infection rate for Method 2 was 6.25% for supratentorial surgeries, but significantly higher at 23.33% for infratentorial surgeries, indicating a significant difference (*P* = 0.039); this difference was primarily driven by a higher incidence of incision infection/delayed healing in the infratentorial group (16.67% vs. 2.08%, *P* = 0.028), whereas meningitis rates were comparable (6.67% vs. 4.17%, *P* = 1.000). The incidence of pseudomeningocele showed no significant difference between supratentorial and infratentorial sites (2.08% vs. 0%). Pooled data analysis revealed a trend toward a higher postoperative infection rate in patients undergoing Method 2 compared to Method 1 (12.82% vs. 4.26%, *P* = 0.057), which approached but did not reach statistical significance. Although the above description shows the probability of outcomes for each method at different sites, multivariable logistic regression analysis (Table 2), after adjusting for confounding factors, confirmed that the surgical site itself was not an independent risk factor for infection or other complications. Specifically, in the fully adjusted model (Model 3), the infratentorial site was not independently associated with increased risk of overall infection (OR 2.49, 95% CI 0.62–11.3, *P* = 0.210), meningitis (OR 1.67, 95% CI 0.21–11.4, *P* = 0.694), incision infection/delayed healing (OR 1.85, 95% CI 0.24–17.0, *P* = 0.554), pseudomeningocele (OR 4.46, 95% CI 0.24–285, *P* = 0.365), or CSF leak (OR 4.46, 95% CI 0.24–285, *P* = 0.893). This further suggests that the high infection rate of Method 2 in the infratentorial region might be related to the inherently higher complexity of the patient population requiring this method. Figure 1 illustrates the two surgical procedures.

**Table 1:** Baseline characteristics and outcomes by surgical site and repair method.

Characteristics	Method 1				Method 2			
	Total (*n* = 94)	Supratentorial (*n* = 42)	infratentorial (*n* = 52)	*P*	Total (*n* = 78)	Supratentorial (*n* = 48)	Infratentorial (*n* = 30)	*P*
Gender, *n* (%)				0.063				0.619
Male	34 (36.17)	20 (47.62)	14 (26.92)		30 (38.46)	20 (41.67)	10 (33.33)	
Female	60 (63.83)	22 (52.38)	38 (73.08)		48 (61.54)	28 (58.33)	20 (66.67)	
Age (mean ± SD)	58.47 ± 13.92	56.52 ± 15.94	60.04 ± 11.97	0.240	56.40 ± 14.59	55.04 ± 14.88	58.57 ± 14.09	0.297
weight (mean ± SD)	65.55 ± 11.34	67.20 ± 10.98	64.22 ± 11.55	0.204	64.93 ± 10.04	66.01 ± 8.69	63.19 ± 11.83	0.265
height (mean ± SD)	161.90 ± 8.39	163.32 ± 8.35	160.75 ± 8.32	0.141	161.97 ± 8.31	163.30 ± 7.69	159.83 ± 8.93	0.083
BMI (mean ± SD)	25.38 ± 4.39	25.33 ± 4.13	25.42 ± 4.63	0.914	24.96 ± 3.52	24.90 ± 2.82	25.06 ± 4.46	0.865
Hypertension, *n* (%)	19 (20.21)	9 (21.43)	10 (19.23)	0.996	21 (26.92)	15 (31.25)	6 (20.00)	0.408
Diabetes, *n* (%)	2 (2.13)	2 (4.76)	0 (0.00)	0.383	4 (5.13)	2 (4.17)	2 (6.67)	1.000
CAD, *n* (%)	3 (3.19)	2 (4.76)	3 (3.19)	0.851	1 (1.28)	1 (2.08)	0 (0.00)	1.000
epilepsy, *n* (%)	1 (1.06)	1 (2.38)	0 (0.00)	0.914	0	0	0	
cerebral infarction, *n* (%)	2 (2.13)	1 (2.38)	1 (1.92)	1.000	2 (2.56)	1 (2.08)	1 (3.33)	1.000
Dura repair time,mean ± SD (min)	23.32 ± 4.46	22.83 ± 4.83	23.71 ± 4.15	0.353	31.86 ± 4.49	31.27 ± 4.54	32.80 ± 4.33	0.141
LD, *n* (%)	20 (21.28)	8 (19.05)	12 (23.08)	0.825	15 (19.23)	9 (18.75)	6 (20.00)	1.00
Type of surgery, *n* (%)				0.729				0.167
Neuro-oncological, *n* (%)	45 (47.87)	22 (52.38)	23 (44.23)		52 (66.67)	34 (70.83)	18 (60.00)	
Neurovascular, *n* (%)	42 (44.68)	17 (40.48)	25 (48.08)		13 (16.67)	9 (18.75)	4 (13.33)	
Others	7 (7.45)	3 (7.14)	4 (7.69)		13 (16.67)	5 (10.42)	8 (26.67)	
pathological pattern, *n* (%)				0.776				0.279
WHOⅠ–Ⅱ	32 (62.75)	14 (58.33)	18 (66.67)		41 (70.69)	24 (66.67)	17 (77.27)	
WHOⅢ–Ⅳ	6 (11.76)	4 (16.67)	2 (7.41)		10 (17.24)	8 (22.22)	2 (9.09)	
other malignancies	4 (7.84)	2 (8.33)	2 (7.41)		2 (3.45)	2 (5.56)	0 (0.00)	
Other benign lesions	9 (17.65)	4 (16.67)	5 (18.52)		5 (8.62)	2 (5.56)	3 (13.64)	
CSF leak, *n* (%)	7 (7.45)	3 (7.14)	4 (7.69)	1.000	3 (3.85)	2 (4.17)	1 (3.33)	1.000
Re-repair, *n* (%)	2 (2.13)	2 (4.76)	0 (0.00)	0.383	2 (2.56)	2 (4.17)	0 (0.00)	0.692
infection, *n* (%)	4 (4.26)	3 (7.14)	1 (1.92)	0.464	10 (12.82)	3 (6.25)	7 (23.33)	0.039
Incision infection/delayed healing, *n* (%)	1 (1.06)	1 (2.38)	0 (0.00)	0.914	1 (2.08)	1 (2.08)	5 (16.67)	0.028
meningitis, *n* (%)	3 (3.19)	2 (4.76)	1 (1.92)	0.851	4 (5.13)	2 (4.17)	2 (6.67)	1.000
Pseudo-meningocele, *n* (%)	2 (2.13)	0 (0.00)	2 (3.85)	0.571	1 (1.28)	1 (2.08)	0 (0.00)	1.000

BMI, body mass index; CAD, cornonary artery disease; CSF, cerebrospinal fluid; LD, lumbar drains.

**Table 2:** Results of logistic regression analysis of surgical location and infection (meningitis and incision infection/delayed healing), pseudo-meningocele and CSF leak.

Model	Infection		Meningitis		Incision infection/delayed healing		Pseudo-meningocele		CSF leak	
Model 1										
Supratentorial	—		—		—		—		—	
Infratentorial	1.15 (0.50, 4.79)	0.462	0.82 (0.16, 3.81)	0.795	2.86 (0.60, 20.3)	0.217	2.23 (0.21, 48.4)	0.517	1.10 (0.30, 4.11)	0.879
Model 2										
Supratentorial	—		—		—		—		—	
Infratentorial	1.68 (0.54, 5.48)	0.370	0.86 (0.16, 4.14)	0.845	3.48 (0.69, 26.1)	0.158	2.89 (0.25, 68.8)	0.413	1.04 (0.27, 3.95)	0.957
Model 3										
Supratentorial	—		—		—		—		—	
Infratentorial	2.49 (0.62, 11.3)	0.21	1.67 (0.21, 11.4)	0.694	1.85 (0.24, 17.0)	0.554	4.46 (0.24, 285)	0.365	4.46 (0.24, 285)	0.893

Model 1 was not adjusted; Model 2 was adjusted for age and gender; Model 3 was adjusted on the basis of Model 2 for BMI, diabetes, hypertension, coronary heart disease, epilepsy, cerebral infarction, surgical methods, dura mater repair time, LD, and disease types.

### 4.2 Literature review

The literature review indicates that autologous fascia is widely used in duraplasty with recognized efficacy. A recent meta-analysis (16) pointed out that compared to non-autologous grafts, autologous grafts more effectively reduce the incidence of postoperative meningitis, pseudomeningocele, and overall infection. Several studies support pericranium (17, 18) or nuchal ligament/fascia (14, 15, 19) safe, economical, and effective options in pediatric or adult posterior fossa surgery. Meanwhile, free temporalis muscle fascia (FTFG) has also been proven to be a good material for skull base reconstruction (20, 21). In expanded endoscopic endonasal surgery (EETS), autologous and non-autologous grafts showed similar success rates in preventing CSF leak (22, 23). One study comparing pericranium and human amniotic membrane found no significant difference in complications (24). Facing special circumstances, autologous fascia can also be very effective, such as satisfactory results using autologous fascia lata for dural reconstruction in patients undergoing decompressive craniectomy (25); it is also a good material choice in cranioplasty (26) and surgeries with large cranial and dural defects, achieving satisfactory repair outcomes (27).

## 5 Discussion

The origin of modern dural suturing techniques can be traced to the pioneering work of Harvey Cushing (28), who first established fundamental principles for neurosurgical procedures, including the crucial step of suturing the dura and galea at closure, an innovation that significantly reduced mortality in brain surgery and laid the foundation for dural suturing techniques. Advancements in micro-neurosurgical techniques have provided reliable support for dural suturing, and research on biomaterials has offered diverse choices. Currently, they can be broadly divided into two categories: primary closure and dural repair material closure (autologous fascia, artificial dura). In the dural repair process, watertight closure is generally achieved using interrupted, continuous, or locked suturing techniques. It is generally believed that the suturing technique itself usually has no significant impact on the outcome (29, 30),while the choice of suture material does affect the result (31) (32). Autologous materials: Harvested from the patient's own tissues (e.g., pericranium, fascia, muscle), they offer excellent biocompatibility and no risk of immune rejection, but harvesting may increase surgical trauma and time. Artificial materials: Do not require additional harvesting, but carry issues such as foreign body reaction, chronic inflammation, or differences in degradation cycles (3, 6, 17, 19), Based on these considerations, we propose that the use of autologous fascia for repair, when feasible, in conjunction with a short-cycle artificial dura mater overlay, can effectively prevent postoperative CSF leakage. Furthermore, if intracranial infection occurs, since the circulating CSF does not contact artificial foreign materials, inflammation can be effectively controlled within a certain period, avoiding the potential need for secondary surgery to remove foreign bodies. A prospective clinical trial conducted by Velnar et al. (33) found that autologous muscle fibers are effective for repairing small dural defects, while larger defects have a higher probability of CSF leak, requiring meticulous dural repair, and suggesting the combined use of artificial dura, autologous fascia, and other materials—a conclusion consistent with our practice.

CSF leak is the most serious and most studied complication after dural repair. Literature reports the incidence of CSF leak in neurosurgery ranges from 0% to 34% (34, 35). Typically, postoperative subcutaneous fluid collection is one characteristic of CSF leak (36, 37), and postoperative persistent positional headache also suggests its occurrence (38, 39), Management usually involves conservative treatment, with a small proportion of patients requiring secondary repair. In this study, both methods controlled the CSF leak rate at a low level (<8%), consistent with rates reported in the literature (16, 37) after adopting meticulous repair strategies. This result confirms that the two methods proposed in this study are both effective in ensuring the integrity and reliability of dural repair. Whether direct suturing or pericranium-assisted suturing, as long as strict watertight closure principles are followed (including locked suturing, biological glue reinforcement, and artificial dura coverage), the key pathway for CSF leak can be effectively blocked. Notably, our analysis of re-repair rates revealed low and statistically comparable rates between supratentorial and infratentorial sites for both Method 1 (4.76% vs. 0%, *P* = 0.383) and Method 2 (4.17% vs. 0%, *P* = 0.692). This finding indicates that, despite the higher infection risk associated with pericranium-assisted suturing in the infratentorial region, the mechanical integrity and reliability of the repair in preventing major CSF leaks necessitating re-operation remained robust, further supporting its utility in managing complex dural defects. Infection after autologous fascia repair is also a frequently discussed topic. One literature summary suggested that homologous fascia materials are the choice with the lowest infection rate (40). Regarding infection, our analysis revealed site-specific differences: Method 2 had a significantly higher infection rate in infratentorial surgeries compared to supratentorial surgeries (23.33% vs. 6.25%, *P* = 0.039). Importantly, this increase was predominantly attributable to incision infection/delayed healing (16.67% vs. 2.08%, *P* = 0.028), whereas the rates of meningitis were not significantly different (6.67% vs. 4.17%). This pattern suggests that the pericranium-assisted technique in the posterior fossa may predispose to localized wound complications rather than direct cerebrospinal fluid track-related infections, potentially attributable to the more challenging wound environment, compromised local vascularity, and intricate drainage dynamics characteristic of infratentorial approaches**.** Although multivariable regression analysis suggested the surgical site itself was not an independent risk factor (fully adjusted OR 2.49, *P* = 0.210 for overall infection), mechanistic explanations may involve the following: 1. The infratentorial space is narrow, and differences in CSF circulation dynamics (e.g., posterior fossa high pressure) may cause continuous tension at the fascia repair site, affecting healing; whereas supratentorial dural tension is lower, potentially offsetting the mechanical disadvantages of fascia repair (19, 34); 2. particularly in a region with higher microbial exposure and poorer vascular supply. Although autologous fascia is widely believed to have good biocompatibility and low immunogenicity (7, 11), some studies have pointed out that the additional trauma from fascia harvesting and micro-gaps at the suture interface could become risk points for infection (33). 3. Pericranium has a relatively poor blood supply and weaker anti-infection capability compared to fascia lata, potentially making it more susceptible to disadvantages in the high-pressure, complex CSF dynamics environment of the infratentorium. In the supratentorium, the infection risk of fascia implantation might be offset by the lower susceptibility of the surgical site (e.g., easier drainage of dead space supratentorially). This finding underscores the complex influence of surgical site on the selection of repair materials.

Our findings show both consistency and complexity with the literature. On one hand, numerous studies (14, 15, 19) have reported the successful application of autologous fascia (including pericranium and cervical fascia) in posterior fossa surgery, supporting its safety and efficacy. On the other hand, our discovery shares similarities with the study by Tahami et al. (24), which also found no significant difference in complication rates when comparing pericranium and human amniotic membrane, suggesting that material choice is not the sole determinant, and surgical technique, patient baseline status, and local anatomical environment may all influence outcomes. Additionally, Jurlina et al. reported good performance of free temporalis muscle fascia in skull base reconstruction (24), indicating that autologous fascia from different sources may have differences in anti-infection capability and healing potential.

Another important finding of this study is that although Method 2 had a higher infection rate in the infratentorium, its performance in preventing CSF leak was stable, suggesting that pericranium remains an effective sealing material in complex infratentorial surgeries, but its use requires weighing the infection risk. This aligns with the concept proposed by Velnar et al. (33) of “combining multiple materials to address different defects,” and also suggests that future efforts could further optimize the fascia harvesting and handling process, such as combining short-cycle artificial dura coverage, to balance sealing ability and anti-infection capability.

This study has several limitations. First, the inherent selection bias and confounding bias of its retrospective design cannot be completely avoided. Although we adjusted for known confounders using multivariable regression, some unmeasured variables (such as precise tumor size, relationship to venous sinuses, extent of electrocautery use, patient preoperative nutritional and immune status) might still influence the results. Second, the sample size, particularly in the infratentorial-Method 2 subgroup (*n* = 30), remains insufficient, potentially leading to overestimation or underestimation of the infection risk and affecting the stability of the results. Third, the study focused on “U-shaped” incisions to ensure homogeneity, but this limits the generalizability of conclusions to other incision types (e.g., the common “Y-shaped” incision in the posterior fossa, supratentorial “+” shaped incisions). Finally, the intraoperative judgment of “significant dural shrinkage,” although standardized by the team and provided with quantitative references, still contains subjective components. Despite these limitations, the findings of this study possess clear clinical implications. They suggest that in supratentorial surgeries, both strategies can be safely chosen based on intraoperative conditions. However, in the infratentorium, especially when anticipating complex, prolonged surgery, if planning to use pericranium-assisted suturing, surgeons need to maintain high vigilance for this potential infection risk and should further strengthen aseptic techniques, consider more adequate drainage, and optimize perioperative antibiotic prophylaxis strategies. More importantly, the “Intraoperative Assessment → Repair Strategy Selection” pathway applied in this study provides a clear, executable framework for dural repair. This decision-making model based on objective assessments (such as the PEEP test and degree of shrinkage) helps reduce variability stemming from reliance solely on personal experience. The customized closure process based on incision type proposed by Ma et al. (41) also demonstrates the potential of similar concepts. Future research should focus on prospective, multicenter designs and incorporate more objective dural assessment indicators (such as intraoperative measurement of defect area). Simultaneously, extending the pathway proposed in this study to validate it for other types of dural incisions is an important step towards promoting comprehensive standardization in dural repair practice.

## 6 Conclusion

This study confirms that for U-shaped dural incisions, the two strategies, direct suturing and pericranium-assisted suturing, are equally effective in preventing CSF leak. However, pericranium-assisted suturing presents a higher infection risk in infratentorial surgeries, indicating that in clinical decision-making, especially for complex posterior fossa surgeries, its superior sealing capability must be carefully balanced against its associated infection risk. The “Intraoperative Assessment → Repair Strategy Selection” pathway presented herein offers a valuable decision-making framework for dural repair.

## Data Availability

The raw data supporting the conclusions of this article will be made available by the authors, without undue reservation.
